# Ion beam-induced bending of TiO_2_ nanowires with bead-like and prismatic shapes

**DOI:** 10.1039/d1ra09122k

**Published:** 2022-02-16

**Authors:** Zhina Razaghi, Dong Yue Xie, Ming-hui Lin, Guo-zhen Zhu

**Affiliations:** Department of Mechanical Engineering and Manitoba Institute of Materials, University of Manitoba 75 Chancellors Circle Winnipeg MB R3T 5V6 Canada Guozhen.Zhu@umanitoba.ca; Center for Integrated Nanotechnologies, MPA Division, Los Alamos National Laboratory Los Alamos NM 87545 USA

## Abstract

Ion beam irradiation is a promising method to manipulate the composition and shape of nanowires. It causes the formation of crystal defects like vacancies and dislocations, and consequently, a volume expansion within the irradiated region, giving rise to the nanowire bending. The bending effect has been extensively discussed within nanowires with different diameters under ion beams with varying energies and ion fluences. However, the behaviors of nanowires with complicated shapes, which may have non-uniform irradiated regions due to the changing angle of incidence and shadowing effect, have remained largely unknown. Herein, the structural changes and bending of TiO_2_ nanowires with both bead-like and prismatic shapes are investigated under a Ga^+^ ion beam. The multi-faceted morphology, and consequently, varying angles of incidence, result in inhomogeneous irradiation and volume expansion. As a result, significant bending is only observed in prismatic nanowires. Since irradiation is confined within the half of nanowires facing the ion beam, the bending of nanowires is reversible by changing the direction of the ion beam. In order to provide insights into the tailoring composition and morphology of nanowires, we anticipate that this finding can establish the beam analog at the nanoscale, the bending of which can be tuned by ion irradiation.

## Abstract 2

TiO_2_ nanowires with prismatic shape, show significant bending under an ion beam, compared to those with a bead-like shape.

## Graphical abstract

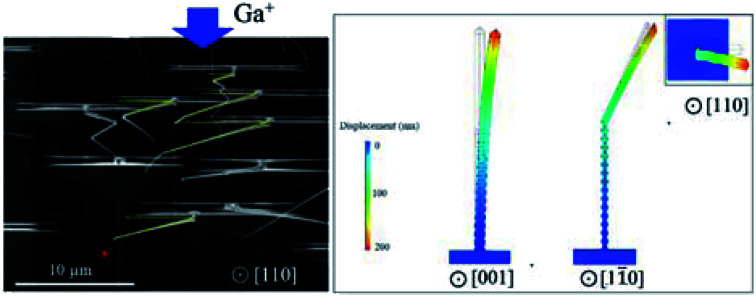

## 1. Introduction

Nanowires with one-dimensional structures and high surface-to-volume ratios have gained much attention in electronic, catalytic, and sensing applications.^1,2^ Semiconductor nanowires used in solar cells significantly reduce reflection, enhance light trapping, improve electron transport and consequently, raise device efficiency, thereby prevailing over their thin-film and nanoparticle counterparts.^3,4^ Originating from their electronic band positions, metal-oxide nanowires hold promise as the favorable base for photoconversion processes, among which rutile titanium oxide (TiO_2_) is a representative candidate for solar cells and lithium-ion batteries applications.^5,6^

The physical and chemical properties of oxide nanowires can be tuned by tailoring their composition and morphology.^7–9^ Ion beam irradiation, during which foreign ions with high energy are implanted into the solid, can manipulate the composition and morphology of the solid.^10–12^ In semiconductor nanowires, ion implantation is widely used as a controllable doping technique, substituting the complex doping during the nanowire growth. For instance, Stichtenoth *et al.*^13^ have demonstrated a manageable route for p-type doping of GaAs nanowires after Zn ion implantation, which leads to an increase in the semiconductor conductivity. Colli *et al.*^14^ have also applied P and B ion implantations in Si nanowires for p- and n-type field-effect transistors. In addition, irradiation with swift heavy ions on copper oxide selenite nanowires shows a change in the plane orientation and also a variation in the electrical resistivity of semiconducting nanowires.^15^ Kwon *et al.*^16^ have illustrated that the gas sensitivity of SnO_2_ nanowires towards NO_2_ increases after He ion bombardment.

The ion irradiation on nanowires can also result in a favorable or even surprising change of morphology due to various atomistic effects. Sputtering, plastic deformation, or compressive and tensile stress are some of these effects which are caused by the collision cascade of ions within the solid. Consequently, structural defects, including vacancies and interstitials, are created, giving rise to the nanowire amorphization.^17–19^ Ion-irradiated amorphization was initially believed to be the essential reason for bending nanowires under an ion beam. Pecora *et al.*^20^ have shown that Si nanowires under Ge ion irradiation with 45 keV and 70 keV energies experience significant bending at a specific point in which the diameter is equal to or less than the amorphization depth. Ion-irradiated amorphization of nanowires occurs under various ion species as a function of ion fluence, and is also linked to the bending of nanowires such as Ge under 30 keV Ga^+^ beam, reported by Romano *et al.*,^21^ Si nanowires under 30 keV Ga^+^ beam, investigated by Jun *et al.*,^22^ and ZnO nanowire under 20–100 keV Ar^+^ beam by Borschel *et al.*^17^ and Ishaq *et al.*^23^

The bending of nanowires has also been observed under ion beams with low fluences and/or low energies. The possible mechanism is that the formation and movement of structural defects such as vacancies and interstitials under ion beams cause local density and volume changes,^24^ instead of amorphization in nanowires. Rajput *et al.*^25^ have discussed that polycrystalline Si nanowires bend towards the incident direction of a 16 keV Ga^+^ ion beam. They reported that compressive stress, induced in the irradiated side of the nanowire caused by sputtering, leads to the nanowire bending towards the ion beam. Ion beams with low energies can also lead to changes in bending directions, which is determined by the irradiation depth regarding the diameter of the nanowire.^17,26^ For instance, Borchel *et al.*^27^ have investigated the bending behavior of GaAs nanowires under the S, Ar, and Xe ions with 20 keV and 100 keV energies. The low-energy ion irradiation causes nanowires to bend away from the ion beam, while high-energy ions lead to an upward bending of nanowires towards the ion beam. The former downward bending is determined by the formation of vacancies and interstitials within the half of the nanowires facing ion beams. On the other hand, the latter upward bending is affected by the deeper ion penetration within the nanowire diameter and surface sputtering under high ion fluence. In addition, the bending of nanowires can alter the incidence angle, a higher value of which causes a higher sputtering and an intense volume change.^28^ For example, Estivill *et al.*^29^ compared different Xe ion ranges at various surface angles of Si nanopillars and showed different sputtering yields at 0°, 45°, and 82.5° angles of incidence.

Among a number of reported studies on ion irradiation of 1D structures, the ion irradiation effect has been largely limited to nanowires with uniform shapes. How the ion beam influences nanowires with complicated shapes is rarely considered. In this work, we investigated the influence of Ga^+^ ion beam irradiation on TiO_2_ nanowires with both bead-like and prismatic shapes. The changes in microstructure were characterized by electron microscopy and then compared to the results from a 3D Monte Carlo simulation. We monitored the bending of nanowires under the Ga^+^ ion beam and compared it to the predicted bending, which was computed by a finite element simulation. Our results provide insights in controlling the morphology of nanowires by ion irradiation and establish a nanoscale beam model for exploiting the bending behavior of nanoscale beams.

## 2. Experimental

### 2.1. Nanowire growth

Rutile TiO_2_ (110) single crystals were purchased from MTI corporation and ultrasonically cleaned with high-purity acetone for 180 minutes. Au–Ag particles were used as the seed to synthesize nanowires with both bead-like and prismatic shapes.^30^ A mixture of gold and silver nanoparticle suspensions with different ratios up to 50 wt% silver concentration was prepared. These nanoparticle suspensions were synthesized by the Turkvich method and they were subsequently dropped and evaporated on oxide substrates. The temperature of the oxide substrate was sustained at 200 °C during particle deposition to avoid the agglomeration of deposited nanoparticles.

The particle-deposited oxide substrate was sealed in a glass vessel, with a diameter of 0.8 cm and a length of 15 cm, which was filled by Ar gas of 99.999% purity. The length of the vessel was chosen according to the isothermal region of the tube furnace, and the vessel was heated in the tube furnace to 1000 °C for 60 min. The rates of heating and cooling were 10 °C min^−1^ and 2 °C min^−1^, respectively.

### 2.2. Ion beam irradiation

In order to investigate the behavior of TiO_2_ nanowires under ion beam irradiation, TEScan FIB-SEM was applied to observe the morphology changes of nanowires under Ga^+^ ion irradiation at 30 keV. A Ga^+^ ion beam with a current of 24 pA was scanned over nanowires. Firstly, an area of ∼ 31.8 × 21.3 μm^2^, with an ion dose of 4.32 × 10^8^ cm^−2^, was scanned to analyze the bending behaviors of nanowires. After that, a sequence of scans over an area of 137.3 × 91.6 μm^2^, with an ion dose of 4.57 × 10^7^ cm^−2^ were repeatedly carried out along two reverse directions, to study the recoverability of the nanowires bending.

In order to study microstructural changes using transmission electron microscopy (TEM), as-synthesized nanowires were single scanned by Ga^+^ ion beams at a fairly large current over the entire samples to ensure a similar dose mentioned above. Subsequently, nanowires were scraped onto Cu grids. A Monte Carlo simulation using IM3D (Irradiated Microstructures in 3D) code was applied to model the distribution of damage in nanowires, under ion irradiation.^31^ A three-dimensional (3D) morphology of nanowires was constructed using Finite Element Triangle Mesh (FETM) and inputted into IM3D. The defect distribution was estimated by full cascade methods. Ga^+^ ions, with the energy of 30 keV, were implanted at an incidence angle of 38° from the surface normal. 50 000 Ga^+^ ions were simulated to obtain valid statistical distribution.

A finite element method was used to qualify possible volume expansion resulted from Ga^+^ ion beam, so that the bending behaviors under the ion beam can be explained. Such volume expansion was estimated using a thermal expansion model, in which the temperature profile was set according to the implantation profiles from Monte Carlo simulations. The finite element simulation was conducted using Ansys software. The 3D model of nanowires was input for the bending simulation. The geometry model of the nanowire and substrate was generated with APDL code. The model was meshed using automatic smart meshing, which ensures sufficient mesh density in the nanowire region. The 20-node solid element SOLID186 was employed in the simulation. During the simulation, the bottom of the substrate was fixed, and the temperature of the irradiated region was increased. The thermal expansion was used to imitate the irradiation swelling. The thermal expansion and temperature values were selected by measuring the simulated bending angle of 13°.

## 3. Results and discussion

**Fig. 1:**
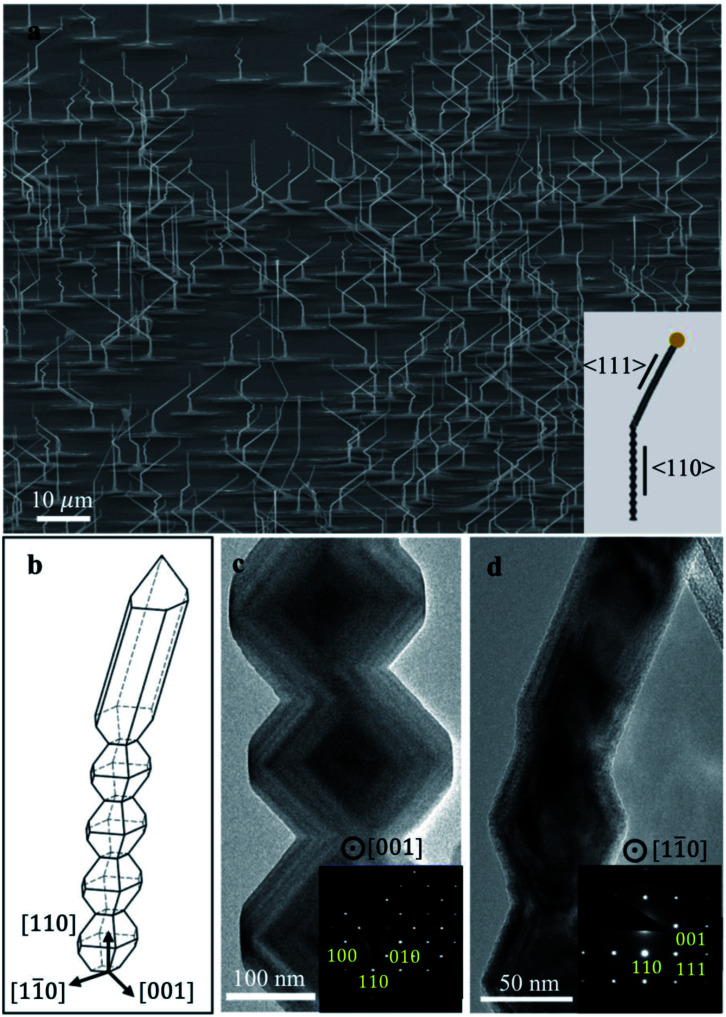
Morphology of TiO_2_ nanowires with Au–Ag bimetallic seeds. (a) Scanning electron microscopy (SEM) image along with schematic graph of the nanowire produced in 〈110〉 and 〈111〉 directions (compatible with the TEM observation). (b) 3D model of the bead-like and prismatic regions, showing different nanowire facets. (c and d) Transmission electron microscopy (TEM) images of bead-like, and prismatic parts of rutile nanowires under [001] and [11̄0] zone axes, respectively.

To investigate the shape effect on the response of nanowires to the ion beam, TiO_2_ nanowires, with both bead-like and prismatic shapes, are synthesized using Au–Ag bimetallic seeds. As shown in SEM images in Fig. 1(a), most nanowires grow vertically at the beginning and then can twist toward a specific direction. Some nanowires change their growth directions repeatedly while only a few nanowires are vertical with respect to the substrate surface (*i.e.*, (110)_TiO_2__). It should be noted that underneath individual nanowires, rectangular pyramid bases are rutile bases and are elongated along 〈001〉_TiO_2__.^32,33^ This feature can be used to indicate the crystallographic directions of nanowires while being observed in SEM.

Nanowires have their growth directions either along 〈110〉_TiO_2__ (bead-like shape, vertical to the substrate) or 〈111〉_TiO_2__ (prismatic shape, inclined to the substrate), as indicated from the TEM bright-field images and corresponding diffraction patterns in Fig. 1(c and d). The vertical nanowires have a bead-like shape enclosed by two {1̄10}_TiO_2__ and eight {101}_TiO_2__ facets, and the inclined nanowires have a prismatic shape consisting of two {11̄0}_TiO_2__ and four {101}_TiO_2__ facets.^30^ Accordingly, the schematic graph of nanowires was plotted in Fig. 1(b), which illustrates the detailed morphology of nanowires. These facets have been reported as low-energy surfaces.^34,35^ The changes in growth directions of nanowires are caused by the presence of Ag-rich segregation within Au–Ag bimetallic seeds.^30^ Prismatic nanowires grow when Ag-rich segregations present at the growing nanowire front, and bead-like nanowires form when Ag-rich segregations are away from the growth front. Ag-rich segregations can change their positions during the growth of nanowies and thus change the growth directions of nanowires.

**Fig. 2:**
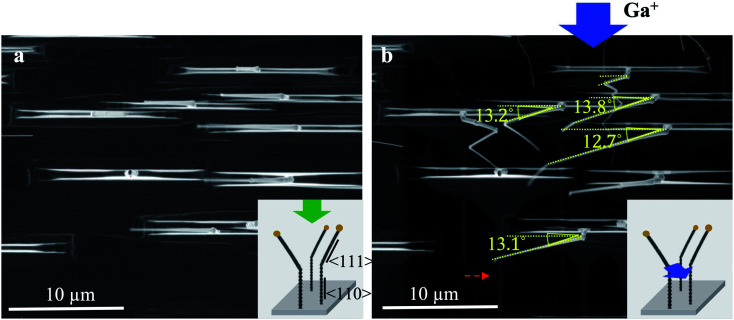
(a) Original twisted nanowires. (b) Bent nanowires after Ga^+^ ion scan along [11̄0]_TiO_2__ direction. The green and blue arrows in the inserted images show the view and beam directions, respectively. The red dashed line indicates shadow images of nanowires due to the scan of Ga^+^ ion beam.

Fig. 2 displays the top view of twisted nanowires before and after Ga^+^ ion beam scan. Viewed along [110]_TiO_2__ (as shown in the green arrow in the schematic graph in Fig. 2(a)), vertical nanowires (along 〈110〉_TiO_2__) are projected as bright dots and twisted parts (along 〈111〉_TiO_2__) are projected along 〈001〉_TiO_2__. The projection of 〈111〉 nanowires is overlapped with the projections of rectangular pyramid bases. After Ga^+^ ion beam scanning, a significant deviation and bending can be detected for 〈111〉-nanowires, as shown in Fig. 2(b). The bending angles for each nanowire are indicated by the yellow dash lines. It can be detected that the bending angles are ∼13° for all observed 〈111〉-nanowires. The bending angles are identical, suggesting a uniform bending effect caused by Ga^+^ ion beams.

Since the electron and ion beams intersect with an angle of ∼52° within the current Focused Ion Beam (FIB) machine, the ion beam is ∼52° off the normal direction to the substrate, that is, 〈110〉_TiO_2__. Thus, the Ga^+^ ion beam is close to 〈100〉_TiO_2__, with ∼7° off toward 〈11̄0〉_TiO_2__, as labeled by the blue arrow in Fig. 2(b). This can also be proved by the shadow image of rutile bases and nanowires as a result of the scan of Ga^+^ ion beam (labeled by the dashed red lines). These bases are ∼2.0–2.5 μm in height, corresponding to the measurable shadow length of ∼2.8–3.1 μm when the ion beam is 38° tilting off the substrate surface.

**Fig. 3:**
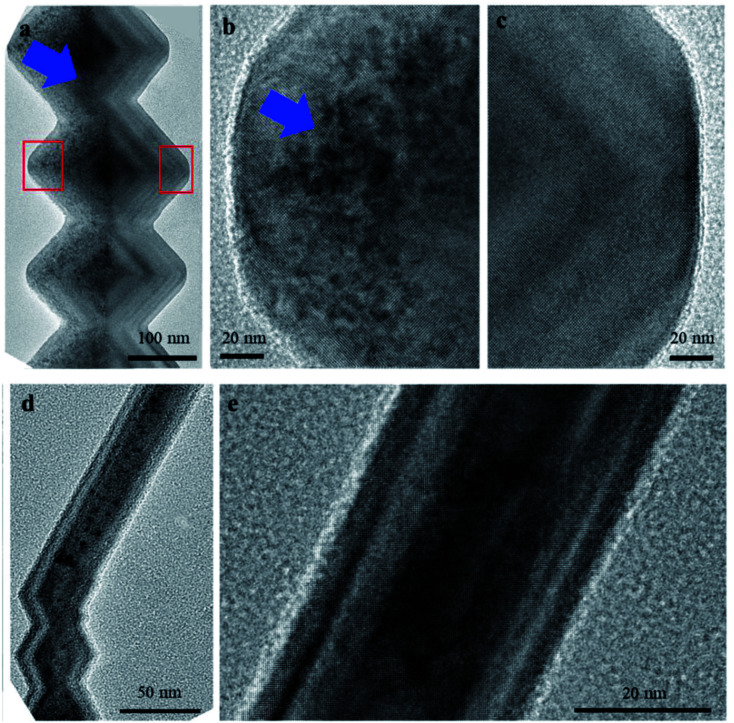
Microstructural changes of TiO_2_ Nanowire after the Ga^+^ ion scan. (a) Heavy ion irradiation in the beam direction (*i.e.*, left part of the nanowire). (b and c) Enlarged images highlighting the ion irradiated and non-irradiated parts, respectively, viewed along [001]_TiO_2__ direction. (d) Irradiation distribution, viewed along the scanning direction (*i.e.*, [11̄0]_TiO_2__), and (e) its magnified image.

TEM observation has been conducted on individual nanowires to clarify the bending of nanowires. Viewed along [001]_TiO_2__, Fig. 3(a) shows the effect of Ga^+^ ion irradiation on the bead-like nanowires. Different contrast appears in the left side of these beads, which includes four {101̄} and one {11̄0} facets facing the ion beam. This observation represents remarkable irradiation damage in this area, stemming from ion-induced vacancies and interstitials. A clear boundary between affected and non-affected regions is detected, distinguishing the facets facing ion beam or not. The irradiated and non-irradiated areas, labeled by red rectangles in Fig. 3(a), are magnified in Fig. 3(b) and (c). Inhomogeneous ion irradiation is detected within the left part of these beads. Ion-irradiated damage, referring to vivid dark regions, is within ∼20 nm to 100 nm from the {11̄0} facets, while the near-edge parts experience less damage. The top half has heavier damage since it faces the Ga^+^ beam, as shown in the blue arrow in Fig. 3(a and b).

On the other hand, ion irradiation appears over the entire bead while viewed along the perpendicular view direction, [11̄0]_TiO_2__, as shown in Fig. 3(d). This is expected that TEM images are two-dimensional projections of three-dimensional samples, as a result, Fig. 3(d) contains accumulated information of three-dimensional ion irradiation profiles, projected along their view directions. It should be noted that the TEM contrast is influenced by the local thickness, which continuously changes from the near-edge region to the center, as shown by changing the contrast in the non-ion-irradiated part of Fig. 3(c). It is expected that only half of the prismatic nanowires have ion irradiation since they consist of the same {11̄0} facet and the two of the four {101} facets of bead-like nanowires. However, prismatic nanowires are rarely viewed along [001]_TiO_2__ and show damages within half of the nanowires. The reason is that these twisted nanowires likely lay on the TEM grids, as a result, 〈110〉_TiO_2__ which is vertical to both bead-like and prismatic nanowires is normal to TEM grids. Therefore, the prismatic part is hardly tilted to be viewed along [001]_TiO_2__. On the other hand, TEM observation along [11̄0]_TiO_2__ indicates relatively uniform ion irradiation, as predicted from the scanning direction of the Ga^+^ ion beam.

**Fig. 4:**
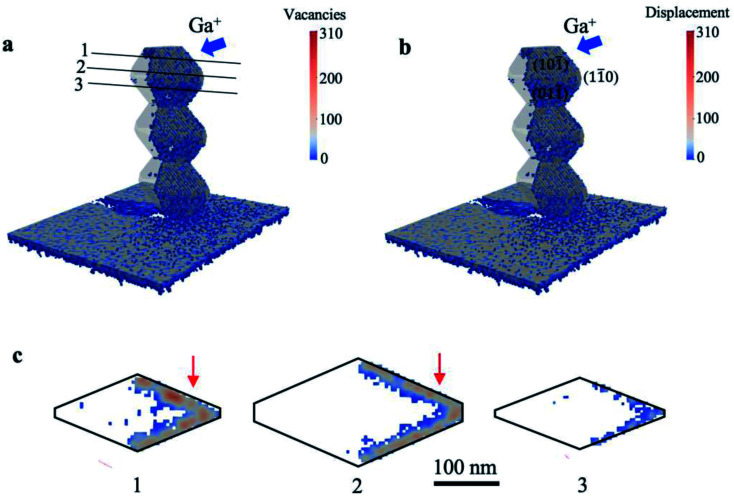
Spatial distribution of (a) vacancies and (b) displacement within nanowire beads. Blue arrows indicate the incoming direction of Ga^+^ ions. (c) Cross-sections of one bead through three different widths sectioned by lines 1, 2, and 3. The red arrows indicate the heavy damage accumulated along [001] within a given depth.

As the indicators of damage profile, the distribution of vacancies and displacements was simulated using the IM3D Monte Carlo code. The simulated results of a bead-like nanowire with a width of ∼300 nm are shown in Fig. 4(a and b). A wide range of defect distribution can be seen in the irradiated region of the beads. The upmost bead shows more damage on its top facet since it faces the ion beam. The shadowing of ions on their incident path by nanowire is detectable on the substrate behind the nanowire. Fig. 4(c) illustrates the cross-sections of one bead as indicated by Sections 1, 2, and 3 to shed light on the damage depth within different facets. It is clear that ion beams concentrate on the surface of each facet and scatter diversely in various nanowire widths. The incidence angles of Ga^+^ ion beam with each facet of {11̄0}, top {101̄}, and bottom {01̄1̄} were calculated to be 38°, 33.4°, and 5.9°, respectively, leading to minor differences in the vacancies and displacement profiles. A high amount of displacement and consequent damage can be observed in {11̄0}, top {101̄} facets. The induced damage in the bottom {01̄1̄} facet is relatively lower, which is aligned with the TEM observation in Fig. 3(a and b). It should be noted that TEM observation is the projections of a 3D object; thus, the observed damage in TEM is accumulated along the view direction. As shown in the red arrows in Fig. 4(c), projected heavier damage appears in the depth of ∼40 nm from the {001} facets. This behavior also agrees with the TEM observation in Fig. 3(a and b).

The distribution of damage stems from Ga^+^ ion interaction with the atomic nuclei and electrons, which leads to slow down by electronic and nuclear stopping. If the energy transferred from the ion beam to the target nucleus is higher than its displacement energy, the nucleus would be knocked off its respective lattice position. A collision cascade would happen by further displacing the nuclei from their lattice sites. Consequently, some places remain unoccupied as vacancies. Displaced atoms can take up interstitial positions, giving rise to volume expansion in the crystalline structure.^20,36^ We hypothesized that such volume expansion causes the detected bending of prismatic nanowires under Ga^+^ ion beam, as shown in Fig. 2. The volume expansion cannot cause significant bending of bead-like nanowires since significant damage appears within protruding areas and cannot lead to continuous volume expansion within the half of the bead-like nanowires. On the other hand, the volume expansion is confined within the half of prismatic nanowires, leading to significant bending of prismatic nanowires.

**Fig. 5:**
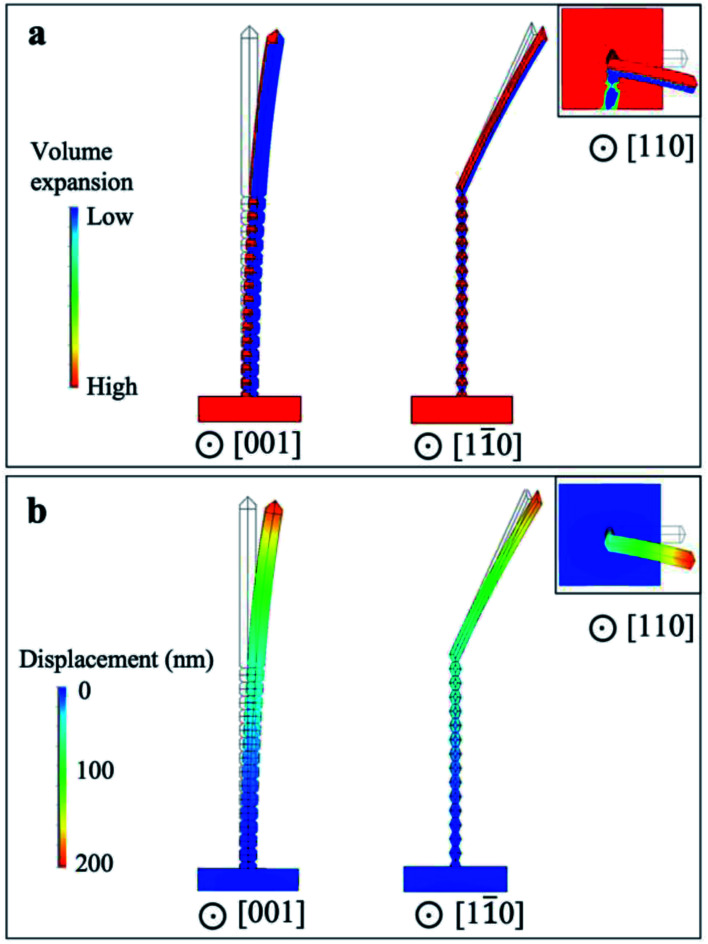
Finite element simulation on possible volume expansion within nanowires and displacement of nanowires. (a) The volume expansion profile and (b) the displacement profile along [001]_TiO_2__ and [11̄0]_TiO_2__ directions. The original nanowires are plotted with black lines and overlapped with irradiated nanowires, which are plotted in color. The inserted image shows the top view.

Accordingly, we have simulated the bending behaviors of nanowires using the finite element method. The expansion of volume is introduced based on a thermal expansion model. As shown in Fig. 5(a), the temperature profiles are set to give an equivalent expansion effect with the simulated ion irradiation profile within the half of the nanowires, with a fixed thermal expansion coefficient. Subsequently, different volume expansions are introduced within different parts of nanowires. Fig. 5(a) and (b) show the simulation results on induced ion irradiated bending of nanowires along [001]_TiO_2__, [11̄0]_TiO_2__, and [110]_TiO_2__ (top view, inserted figure), respectively. The original morphology of nanowires is plotted by black lines and is overlapped with the simulated morphology of nanowires, the color of which indicates the volume expansion profile in Fig. 5(a) and displacement profile in Fig. 5(b). Viewed along [001]_TiO_2__, the volume expansion concentrates in the left parts of the nanowire, which has been irradiated by the ion beams. The right side of the nanowire is shadowed from its left part; as a result, no ion-irritated damage and no volume expansion are considered. The prismatic nanowires exhibit a 13° off the original positions, mimicking the bending response detected under the Ga^+^ ion beam. It should be noted that prismatic nanowires slightly bend towards the substrate, viewed along [11̄0]_TiO_2__. This is reasonable since the ion beam is 13° off the substrate surface. Such downward bending cannot be observed from the top-view images in Fig. 2.

**Fig. 6:**
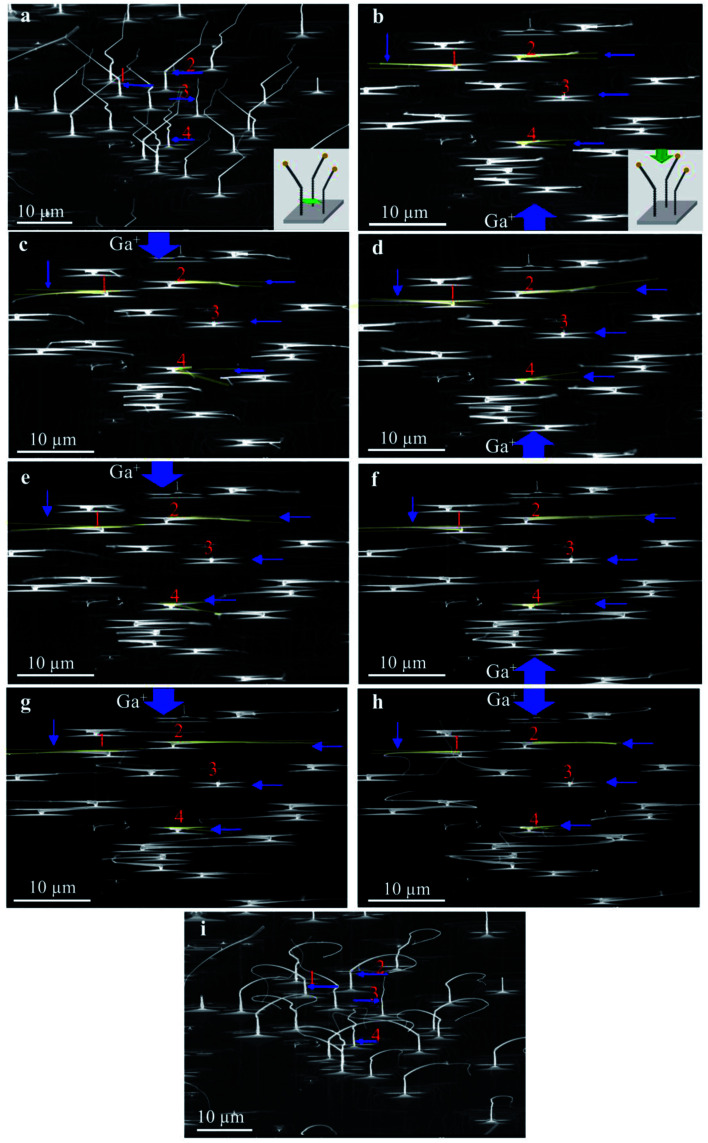
(a) Tilted view with 20° angle of original twisted nanowires, including vertical nanowires (along [110]_TiO_2__ direction). The schematic image indicates the beam track in the side view [001]. Top view of the twisted nanowires after (b) first Ga^+^ ion scan along [11̄0]_TiO_2__ direction (c) second Ga^+^ ion scan along [1̄10]_TiO_2__ direction and again (d) third (e) fourth (f) twenty fifth (g) fiftieth and (h) one hundred times along the given ion scan directions, respectively. The schematic image indicates the beam track in the top view. (i) Tilted view of nanowires after 100 scan times.

Since ion irradiation has been carried out with relatively low fluences at room temperature, the induced collision cascade can be partially recovered, and therefore, the bending may be reversed under opposite ion beams. Accordingly, Ga^+^ ions are scanned back and forth 100 times; the results are summarized in Fig. 6. Fig. 6(a) shows original twisted nanowires, including vertical nanowires (along [110]_TiO_2__) and prismatic parts (along [111]_TiO_2__) through the tilted view, as given in the inserted schematic figure (the green arrow shows the observing direction). A tilted view of nanowires after 100 times scan in Fig. 6(i) demonstrates bending occurring in the prismatic region with a relatively high curvature while no significant change in the bead-like part of the nanowires can be observed. Such high curvature is reasonable since the beam is 38° off the substrate surface, resulting in heavy damage in the top half of nanowires. Accordingly, it leads to additional bending slowly toward the substrate surface. Such downward bending is also predicted by the finite element simulation in Fig. 5, and is accumulated after scans along both directions.

The small blue arrows with the corresponding numbers in Fig. 6(b–h) highlight specific nanowires bent in two reverse directions. We selected these four nanowires with different morphology to compare their bending behavior. According to Fig. 6(a), NW1 consists of one vertical segment and one twisted segment. NW2 twists two times and has three distinct parts. Also, NW3 and NW4 represent an almost straight nanowire and the one with several twisting directions, respectively. After the reverse scans, nanowires bent in the reverse direction, from the comparison between Fig. 6(c) to (b). It should be noted that the downward bending may cause violent curvature of nanowires after multiple scans, and can be more significant near the tip.

It was revealed that the angle of NW3 did not change after ion irradiation, as can be detected in the top view images (Fig. 6(c–h)) and also by comparing the tilted view ones (Fig. 6(a) to (i)). For NW1, NW2, and NW4, prismatic nanowires bend intensely after the first scan, while the bending angle would gradually decrease by increasing the scan sequence. The reason is that significant damage and subsequent bending are introduced by the first scan. Such damage can be partially recovered. The same amount of damage, which is introduced by the following reverse scan on the opposite half of nanowires, causes the reverse bending at a smaller angle. This bending angle would diminish as reverse scans continue to repeat because of the residual volume expansion within the nanowire in the reverse orientation.

## 4. Conclusion

In summary, the effect of Ga^+^ ion beam on the TiO_2_ nanowires with bead-like and prismatic shapes has been studied. The ion beam results in an inhomogeneous deformation, agreeing the computed results from Monte Carlo simulation. Such ion irradiation causes the volume expansion, which can be released within bead-like nanowires. In contrast, the induced volume expansion gives rise to the bending of prismatic nanowires. The bending behavior is supported by the Finite element simulation. In addition, while the bending behavior is reversed by ion irradiation in the opposite direction, it cannot be recovered entirely due to the residual damage within the nanowire. These results propose a nanoscale beam model for bending, considering the nanowires shape and growth direction.

## Author contributions

Z. Razaghi performed data analysis and wrote the manuscript. D. Y. Xie carried out the experimental and Finite element simulation. M.-h. Lin contributed in data analysis. G.-z. Zhu supervised the project. All authors discussed the results and contributed to the final manuscript.

## Conflicts of interest

The authors declare that they have no known competing financial interests or personal relationships that could have appeared to influence the work reported in this paper.

## Supplementary Material
